# An Adaptive Weighted Residual-Guided Algorithm for Non-Uniformity Correction of High-Resolution Infrared Line-Scanning Images

**DOI:** 10.3390/s25051511

**Published:** 2025-02-28

**Authors:** Mingsheng Huang, Weicong Chen, Yaohua Zhu, Qingwu Duan, Yanghang Zhu, Yong Zhang

**Affiliations:** 1 University of Chinese Academy of Sciences, Beijing 100049, China; huangmingsheng@mail.ustc.edu.cn (M.H.); chenweicong@mail.sitp.ac.cn (W.C.); zhuyaohua@mail.sitp.ac.cn (Y.Z.); zhuyanghang22@mails.ucas.ac.cn (Y.Z.); 2 Shanghai Institute of Technical Physics, Chinese Academy of Sciences, Shanghai 200083, China; duanqw2023@shanghaitech.edu.cn; 3 School of Information Science and Technology, ShanghaiTech University, Shanghai 201210, China

**Keywords:** infrared line-scanning images, non-uniformity correction, residual-guided filtering, adaptive weighted linear regression

## Abstract

Gain and bias non-uniformities in infrared line-scanning detectors often result in horizontal streak noise, degrading image quality. This paper introduces a novel non-uniformity correction algorithm combining residual guidance and adaptive weighting, which achieves superior denoising and detail preservation compared to existing methods. The method combines residual and original images in a dual-guidance mechanism and significantly enhances denoising performance and detail preservation through iterative compensation strategies and locally weighted linear regression. Additionally, the algorithm employs local variance to adjust weights dynamically, achieving efficient correction in complex scenes while reducing computational complexity to meet real-time application requirements. Experimental results on both simulated and real infrared datasets demonstrate that the proposed method outperforms mainstream algorithms regarding peak signal-to-noise ratio (PSNR) and structural similarity (SSIM) metrics, achieving an optimal balance between detail preservation and noise suppression. The algorithm demonstrates robust performance in complex scenes, making it suitable for real-time applications in high-resolution infrared imaging systems.

## 1. Introduction

In recent years, significant advancements in infrared imaging technology and manufacturing processes have led to widespread application in thermal imaging, environmental monitoring, and military operations [1,2]. However, infrared detectors are susceptible to environmental temperature and voltage fluctuations, which cause variations in response coefficients. These variations generate fixed-pattern streak noise that is difficult to eliminate through hardware solutions, thereby degrading image quality and adversely affecting subsequent image processing [3,4,5,6].

Researchers have developed non-uniformity correction methods to address these challenges, mitigate streak noise, and improve image quality. Non-uniformity correction methods can be broadly classified into reference-based [7] and scene-based approaches [8,9]. Reference-based correction methods use blackbody images to calculate correction coefficients [10,11,12,13,14,15,16]. While straightforward and easy to implement, these methods have notable limitations. They require periodic calibration, which is particularly inconvenient in dynamic environments where calibration coefficients can drift over time as operating conditions change [13]. Recent studies have attempted to address thermal drift via a semi-transparent shutter [17] or camera housing stabilization [18]. However, such hardware-based solutions can still be challenging for real-time or large-scale deployments. Moreover, standard global calibrations may be inadequate in fine-detail scenarios, as Pron and Bouache [19] show that localized or pixel-level calibration can outperform manufacturer-provided corrections, albeit with increased computational overhead. Furthermore, reference-based methods struggle with complex noise patterns, especially in variable environments, and fail to provide effective real-time solutions.

Unlike reference-based methods, scene-based correction methods do not rely on external reference images and include correction algorithms based on filtering, statistics, model optimization, and neural networks. Despite their promise, scene-based methods face several challenges. Filter-based methods effectively remove low-frequency noise but often cause significant detail loss, particularly in high-resolution images [20,21,22,23,24,25,26,27]. Optimization-based methods, which leverage techniques such as total variation regularization or low-rank matrix decomposition, can effectively separate noise but suffer from high computational complexity, making them less practical for real-time high-resolution image processing [28,29,30,31,32,33]. Neural network-based methods excel in noise suppression and image enhancement but require extensive training datasets and computational resources, limiting their applicability [34,35,36,37]. Statistical methods, such as histogram and moment matching, infer noise distribution based on grayscale statistics [38,39,40,41,42]. However, their performance diminishes when noise deviates from assumed distributions, and they often fail to preserve image details and structural features.

High-resolution infrared line-scanning images present unique challenges due to their directional and structured noise, which arises from the same detector element generating each row of pixels. To tackle these challenges, this paper introduces an innovative non-uniformity correction algorithm based on residual guidance and adaptive weighting. The key contributions of this work are as follows:

(1) Residual-guided filtering and dual-guidance mechanism: The proposed method combines residual and original images as guidance for detail preservation and global smoothing, respectively. Through weighted fusion, it addresses the edge-blurring issues inherent in traditional guided filtering, significantly enhancing detail preservation, particularly in high-resolution images.

(2) Iterative residual compensation scheme: A dynamic residual compensation mechanism is introduced to optimize the correction results by gradually smoothing the Gaussian filtering, eliminating the residual noise while avoiding introducing artifacts by over-compensation. Compared with the static compensation scheme in the traditional method, the dynamic compensation can adaptively adjust the compensation intensity according to the distribution of noise, which significantly improves the robustness of the algorithm.

(3) Weighted linear regression based on local variance: Local variance is used to adjust the weights of each region dynamically, and differentiated correction is implemented for areas with different noise intensities, improving global correction accuracy. Unlike the traditional method that uses uniform weights, this algorithm can adaptively adjust according to the local features of the image, which is especially suitable for infrared image processing in complex scenes.

(4) Efficiently adapting to complex scenes: A local image interception strategy based on scene complexity analysis is proposed to optimize computational efficiency, enabling the algorithm to meet the real-time processing requirements in high-resolution images. Compared with the correction method based on region segmentation, this algorithm can characterize the noise distribution of the whole image more accurately without introducing apparent deviations.

Experimental results show that the proposed algorithm achieves excellent denoising and detail preservation under varying noise intensities and complex scenes. Compared with mainstream methods such as those implemented by Cao [43], Li [23], and Ahmed [24], the algorithm significantly improves PSNR and SSIM metrics and demonstrates robust performance in high-resolution infrared line-scanning images. By effectively addressing the limitations of detail preservation and the high computational costs of existing methods, this algorithm provides an efficient and practical solution for non-uniformity correction in complex scenes within infrared line-scanning systems.

## 2. Materials and Methods

**Figure 1:**
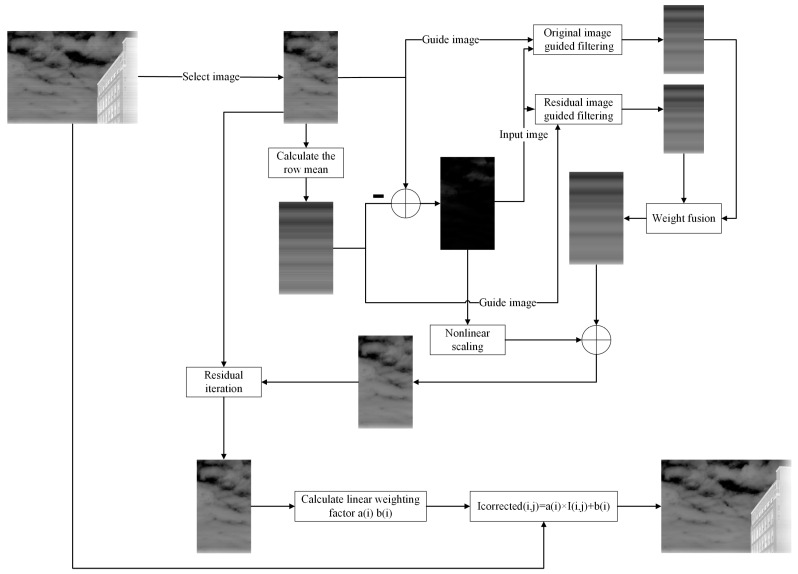
Overall workflow of proposed algorithm.

Infrared line-scanning imaging is characterized by its one-dimensional progressive scanning mechanism, making horizontal stripe noise one of its significant challenges. This noise, caused by the non-uniform response of the detector array, manifests as stripe-like intensity fluctuations along the scanning direction, severely degrading image quality. To address this issue, we propose an innovative residual-guided adaptive correction algorithm. The overall flow of the method is illustrated in Figure 1. 

**Figure 2:**
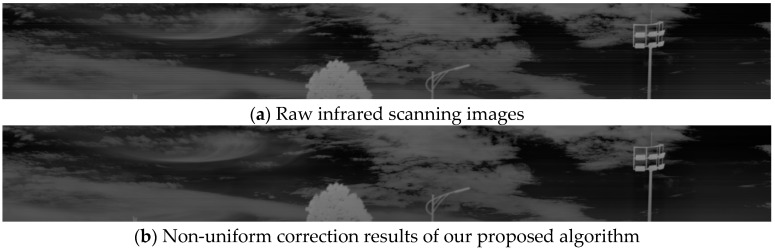
The effect of non-uniform correction of real IR scanned images.

The algorithm comprises four main stages: (1) Row-mean calculation and residual generation: a portion of the image columns is extracted, and the row mean is calculated to generate a residual image. (2) One-dimensional guided filtering and result fusion: The residual and the original images are utilized in a dual-guidance mechanism to perform one-dimensional guided filtering on the row-mean image. Local variance information is then used to fuse the filtering results, producing a preliminary corrected image. (3) Iterative residual compensation: residuals from the filtered image are iteratively compensated using a dynamic optimization approach, refining the corrected result. (4) Linear correction coefficient application: linear correction coefficients are calculated based on the optimized portion of the image and applied globally to correct the entire image. The non-uniformity correction results of the proposed algorithm for real infrared scanning images are shown in Figure 2.

### 2.1. Row-Mean Calculation and Residual Generation

Let the input 2D grayscale image be denoted as $I^{\prime }\left(i,j\right)$. A subset of columns is extracted, forming a smaller grayscale image containing M rows and N columns. The row mean, which represents the average pixel value of all columns in each row, is defined as follows:

(1) $$\bar{I}\left(i\right)=\frac{1}{N}\sum_{j=1}^{N}I\left(i,j\right)$$

where $i\in \left[1,M\right]$ is the row index. The row-mean image $\bar{I}\left(i,j\right)$ is obtained by expanding the mean value of each row across the entire row:

(2) $$\bar{I}\left(i,j\right)=\bar{I}\left(i\right),y\in \left[1,N\right]$$

The row-mean image $\bar{I}\left(i,j\right)$ captures the low-frequency components of stripe noise, while high-frequency details are retained in the residual image, which is defined as follows:

(3) $$R\left(i,j\right)=I\left(i,j\right)-\bar{I}\left(i,j\right)$$

**Figure 3:**
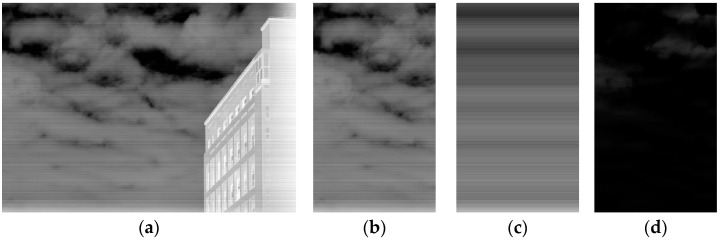
Residual image generation (**a**) original image; (**b**) intercepted image; (**c**) extended row-mean image; (**d**) residual image of an intercepted image.

This operation separates low-frequency noise from high-frequency details, with the residual image as a guide for subsequent filtering. Figure 3 illustrates the residual generation process.

### 2.2. One-Dimensional Guided Filtering and Fusion

#### 2.2.1. Principle of One-Dimensional Guided Filtering

Kaiming He [44] proposed the guided filtering model in 2013. This model structurally optimizes the input image through a local linear model. The key formulas are shown in Equations (4)–(7), and the specific derivation is detailed in Reference [44].

(4) $$q_{i}=a_{k}I_{i}+b_{k},\forall i\in w_{k}$$

In Equation (4), $q_{i}$ is the output image pixel value, $\left(a_{k},b_{k}\right)$ is the linear coefficient within a small window, $I_{i}$ is the guided image pixel value, and $w_{k}$ is the window of the guided filter. Some differences between the output image q and the input image p represent noise or other textures in the image that need to be eliminated. We define the loss function between image p and image q within the window $w_{k}$ as shown in Equation (5):

(5) $$E\left(a_{k},b_{k}\right)=\sum_{i\in w_{k}}\left({\left(a_{k}I_{i}+b_{k}-p_{i}\right)}^{2}+\varepsilon a_{k}^{2}\right)$$

In Equation (5), ε is the penalty parameter. The main goal of guided filtering is to minimize the difference between the output image q and the input image p. The optimal coefficients $a_{k}$ and $b_{k}$ are given by the following equations:

(6) $$a_{k}=\frac{\frac{1}{\left|w\right|}\sum_{i\in w_{k}}I_{i}p_{i}-\mu_{k}\bar{P_{k}}}{\frac{1}{\left|w\right|}\sum_{i\in w_{k}}\sigma_{k}^{2}+\varepsilon }$$

(7) $$b_{k}={\bar{p}}_{k}-a_{k}\mu_{k}$$

where $\mu_{k}$ and $\sigma_{k}^{2}$ are the mean and variance of I within $w_{k}$, $\left|w\right|$ is the number of pixels within the window $w_{k}$, and ${\bar{p}}_{k}$ is the mean of the input image $p_{k}$ within the window $w_{k}$, respectively.

#### 2.2.2. Image-Guided Process

Guided filtering typically uses a single guide image, which can lead to trade-offs between preserving details and achieving smoothness [44]. To overcome this, our method introduces a dual-guidance approach, using both the residual and original images as guides. The residual image captures fine details by focusing on high-frequency noise, while the original image provides overall structural information to ensure large-scale consistency. By combining these two guides, the algorithm achieves enhanced detail preservation and noise suppression. The residual image R(i,j) isolates high-frequency details for effective low-frequency noise optimization. In contrast, the original image contains global structural information, which enhances large-scale smoothing and edge preservation.

**Figure 4:**
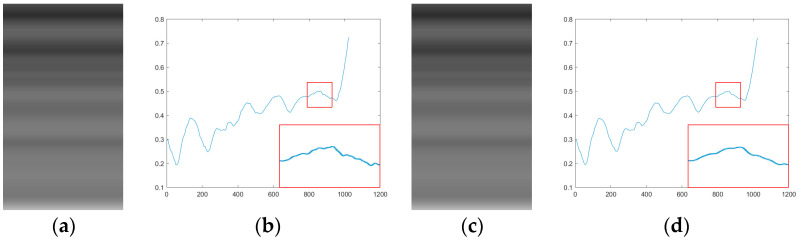
Results of one-dimensional guided filtering using dual guidance. (**a**) Corrected image using residual image as guide; (**b**) signal map derived from (**a**) shows preserved local variations and improved texture fidelity; (**c**) corrected image using original image as guide; (**d**) signal map derived from (**c**) highlights smoother transitions and large-scale noise suppression.

Based on the principle of guided filtering described in Section 2.2.1, the residual image $R\left(i,j\right)$ is first used as the guide image. The residual image $R\left(i,j\right)$ which contains detailed signals after stripe noise removal is applied to optimize the row-mean image $\bar{I}\left(i,j\right)$ in a targeted manner. The result of the guided filtering based on the residual image is expressed as follows:

(8) $${\hat{I}}_{R}\left(i,j\right)=a_{R}\left(i\right)R\left(i,j\right)+b_{R}\left(i\right)$$

where $a_{R}\left(i\right)$ and $b_{R}\left(i\right)$ are the local linear coefficients calculated based on the residual image and ${\hat{I}}_{R}\left(i,j\right)$ represents the output image obtained after guided filtering of the row-mean image using the residual image. The results of ${\hat{I}}_{R}\left(i,j\right)$ and the corresponding row-mean signal are shown in Figure 4a,b.

Then, the intercepted original image $I\left(i,j\right)$ is used as the guide image. The original image $I\left(i,j\right)$, which contains global structural and intensity information, enhances global consistency and edge preservation during the guided filtering of the row-mean image $\bar{I}\left(i,j\right)$. The result of guided filtering based on the original image is expressed as follows:

(9) $${\hat{I}}_{I}\left(i,j\right)=a_{I}\left(i\right)I\left(i,j\right)+b_{I}\left(i\right)$$

where $a_{I}\left(i\right)$ and $b_{I}\left(i\right)$ are the local linear coefficients calculated based on the original image. ${\hat{I}}_{I}\left(i,j\right)$ represents the output image obtained after guided filtering of the row-mean image using the original image. The results of ${\hat{I}}_{I}\left(i,j\right)$ and the corresponding row-mean signal are shown in Figure 4c,d.

As observed in Figure 4b, the image exhibits more detailed changes and local fluctuations. In contrast, Figure 4d demonstrates relatively smoother curves, highlighting the preservation of the global structure.

#### 2.2.3. Fusion of Weights

Local variance is employed as the fusion weight to balance the contributions of the two guide images. The local variance reflects the degree of variation in pixel values within a region, representing the texture complexity of the image. The fusion result, denoted as ${\hat{I}}_{fused}\left(i,j\right)$, is expressed as follows:

(10) $${\hat{I}}_{fused}\left(i,j\right)=\omega \left(i,j\right){\hat{I}}_{R}\left(i,j\right)+\left(1-\omega \left(i,j\right)\right){\hat{I}}_{I}\left(i,j\right)$$

where the weight function $\omega \left(i,j\right)$ is defined as follows:

(11) $$\omega \left(i,j\right)=\frac{1}{1+{\mathrm{e}}^{-k\left(\sigma \left(i,j\right)-t\right)}}$$

where k controls the steepness of the mapping and t is the threshold that determines the value at which the local features cause the weights to change rapidly. The computation of $\omega \left(i,j\right)$ depends on the local variance derived from the input image. During the fusion of the two guided filter outputs, a nonlinear sigmoid function is applied to map the local variance and dynamically adjust the fusion weights. Figure 1 illustrates the overall workflow of the residual-guided filtering algorithm.

### 2.3. Nonlinear Scaling and Residual-Based Iterative Compensation

#### 2.3.1. Nonlinear Scaling

The algorithm incorporates a nonlinear scaling strategy before the iterative process to optimize correction results and provide better initial conditions for subsequent iterative residual compensation. This nonlinear scaling is based on the statistical properties of local regions, dynamically adjusting the contribution of residuals to the correction results. The scaling factor is decreased for high-variance regions to prevent over-enhancement of existing details and increased for low-variance regions to enhance potential information. This adjustment refines the distribution of the corrected image, improving both the stability and convergence speed of the iterative compensation.

The Tanh function calculates the final nonlinear scaling factor $S\left(i,j\right)$. Characterized by its steep response within the output range [−1, 1], the Tanh function is sensitive to changes in local variance, making it well-suited as a scaling factor. After transformation, it smooths transitions within the image to prevent artifacts caused by abrupt weight changes. The expression for the nonlinear scaling factor $S\left(i,j\right)$ is given as follows:

(12) $$S\left(i,j\right)=1-\tanh\left(\gamma \left(V\left(i,j\right)-t\right)\right)$$

where $V\left(i,j\right)$ represents the local variance, which reflects the intensity of details in the local region of the image. The parameter t is the threshold for local variance, and γ controls the steepness of the scaling function.

#### 2.3.2. Iteration Based on Residual Compensation

The residual $R_{k}\left(i,j\right)$, representing the difference between the input image and its corrected version after the k-th iteration, is calculated as follows:

(13) $$R_{k}\left(i,j\right)=I\left(i,j\right)-I_{k}\left(i,j\right)$$

where $I\left(i,j\right)$ is the original input image and $I_{k}\left(i,j\right)$ is the corrected image at iteration k.

To achieve optimal calibration, the compensation factor $\alpha_{k}$ is dynamically adjusted. The initial factor $\alpha_{0}$ sets the intensity of the first correction, while subsequent factors decrease with the residual standard deviation $\sigma_{k}$, ensuring stable convergence and avoiding artifacts. The compensation factor is expressed as follows:

(14) $$\alpha_{k}=\alpha_{0}\cdot \left(\frac{\sigma_{k}}{\sigma_{0}}\right)\cdot \beta^{k-1}$$

where $\sigma_{0}$ and $\sigma_{k}$ are the standard deviations of the initial and current residuals, and $\beta \in \left(0,1\right)$ controls the reduction rate.

To suppress high-frequency noise, the residuals $R_{k}\left(i,j\right)$ are smoothed using a Gaussian filter, and the corrected image is updated as follows:

(15) $$I_{k+1}\left(i,j\right)=I_{k}\left(i,j\right)+\alpha_{k}R_{k}^{Smooth}\left(i,j\right)$$

where $R_{k}^{Smooth}\left(i,j\right)$ denotes the smoothed residuals. The iteration stops when the residual standard deviation $\sigma_{k}$ drops below $\varepsilon \sigma_{0}$ (with ε as a predefined scale factor) or when the maximum iteration count $K_{max}$ is reached.

### 2.4. Weighted Linear Regression and Full Map Correction

The algorithm effectively reduces cross-stripe non-uniformity noise in infrared line-scanning images through the above process. However, the high imaging speed of line-scanning detectors—capable of producing tens of thousands of image columns per second—introduces computational challenges. Specifically, the need to calculate linear coefficients for each window $\left(a_{k},b_{k}\right)$ and perform iterative residual compensation increases the algorithm’s runtime, making real-time performance challenging to achieve.

The noise exhibits strong row-wise correlations since the same detector element generates each row in a line-scanning image. To address this, linear correction coefficients a(i) and b(i) are computed for each row to correct the image globally. Traditional uniform-weighted linear regression often leads to under-correction or over-smoothing in regions with varying noise intensities. To mitigate this, the proposed method employs locally weighted linear regression, which dynamically adjusts weights based on local variance.

For a particular row  i, the pixel values in the intercepted image can be expressed as follows:

(16) $$j_{j}=a\left(i\right)*i_{j}+b\left(i\right)+\varepsilon_{j}$$

where a(i)  and b(i) are the linear correction coefficients and $\varepsilon_{j}$ is the fitting error. To reflect the contribution of different pixels to the correction, the weights ω(i, j) are defined as follows:

(17) $$\omega \left(i,\;j\right)=\frac{1}{1+V\left(i,\;j\right)}$$

where V(i, j) represents the local variance, reflecting the noise level.

**Figure 5:**
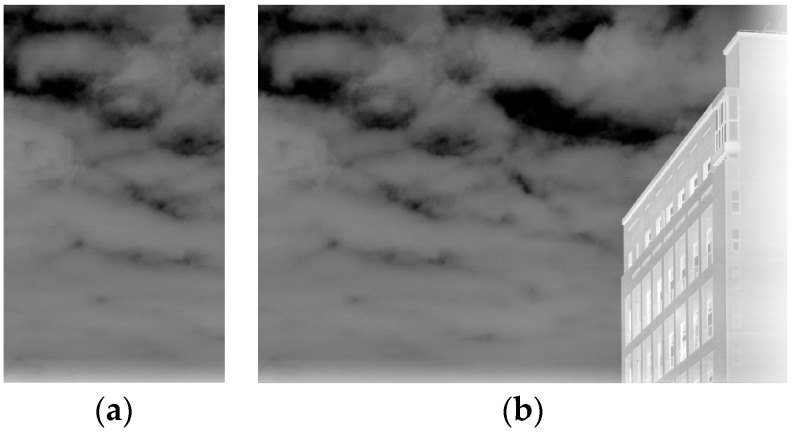
Linear weighted fitting plot. (**a**) Image after residual iteration; (**b**) fitted corrected image.

The linear correction parameters a(i) and b(j) are calculated as Equations (18) and (19), respectively.

(18) $$a\left(i\right)=\frac{\sum_{j}\omega \left(i,\;j\right)I\left(i,\;j\right)I_{k+1}\left(i,\;j\right)-\bar{\omega }\left(i\right)\bar{I}\left(i\right){\bar{I}}_{k+1}\left(i\right)}{{\sum_{j}\omega \left(i,\;j\right)\left({I\left(i,\;j\right)}^{2}-\bar{\omega }\left(i\right)\bar{I}\left(i\right)\right)}^{2}}$$

(19) $$b\left(i\right)={\bar{I}}_{k+1}\left(i\right)-a\left(i\right)\bar{I}\left(i\right)$$

where $\bar{\omega }\left(i\right)=\sum_{j}\omega \left(i,\;j\right)\;$ is the sum of weights and $\bar{I}\left(i\right)$ and ${\bar{I}}_{k+1}\left(i\right)$ are the weighted mean values, respectively. After calculating the correction factors $a\left(i\right)$ and $b\left(i\right)$ for each row, the corrected whole image $I_{corrected}\left(i,j\right)$ can be expressed as follows:

(20) $$I_{\;corrected}\left(i,\;j\right)=a\left(i\right)I^{\prime }\left(i,j\right)+b\left(i\right)$$

where $I^{\prime }\left(i,j\right)$ is the high-resolution input image, and $I_{corrected}(i,\;j)$ is the final corrected image. The corrected result is shown in Figure 5b.

## 3. Results

Our algorithm was compared with several state-of-the-art methods for infrared detectors to evaluate the effectiveness of the proposed non-uniformity correction algorithm. The compared algorithms included the one-dimensional guided filtering algorithm for low-texture infrared images (1DGF) proposed by Cao in 2016 [22], the multi-stage wavelet transform and guided filtering-based denoising algorithm (MSGF) proposed by Cao in 2018 [43], the improved mixed-noise removal method based on non-local means (CNLM) proposed by Li in 2019 [39], the non-uniformity correction method combining one-dimensional guided filtering and linear fitting (GFLF) proposed by Li in 2023 [23], and the stripe noise removal algorithm for one-dimensional signals based on 2D-to-1D image conversion (ENSI) proposed in 2023 [24]. The dataset used in this study comprises six parts: the FLIRADAS dataset [45], the MassMIND dataset [46], Tendero’s dataset [38], the KAIST dataset [47], the LLVIP dataset [48], and long-wave infrared weekly swept real line-scanning images. Experiments were conducted on a 12th generation Intel^®^ Core™ i7-12700H CPU @ 3.61 GHz system with 32 GB RAM and a 64-bit Windows operating system. The software platform used for algorithm implementation was Matlab 2024a.

### 3.1. Noise Modeling Analysis

Unlike array detectors, where each pixel operates independently, infrared line-scanning detectors use the same pixel to scan each row, resulting in each image being generated by the same detector element. This imaging mechanism introduces unique noise characteristics, mainly horizontal streak artifacts. The primary sources of non-uniformity include variations in pixel response and random noise during signal readout.

A noise model comprising gain, bias, and random noise is typically constructed to model non-uniformity and evaluate the effectiveness of the correction algorithm. Changes in gain and bias are modeled as Gaussian random variables: the gain $g\left(i\right)$ at row I follows a Gaussian distribution with a mean of 1 and variance $\sigma_{g}^{2}$, and the bias b(i) follows a Gaussian distribution with a mean of 0 and variance $\sigma_{b}^{2}$. In addition, random white noise $n\left(i,j\right)$ is modeled as a Gaussian distribution with a mean of 0 and variance $\sigma_{white}^{2}$. Combining these factors, the simulated image with noise can be represented as follows:

(21) $$I_{noies}\left(i,j\right)=g\left(i\right)\cdot I_{1}\left(i,j\right)+b\left(i\right)+n\left(i,j\right)$$

where $I_{1}\left(i,j\right)$ denotes the ideal image without noise and $n\left(i,j\right)$ represents the random white noise affecting each pixel.

### 3.2. Effect of Image Size on Non-Uniformity Correction

This experiment analyzes how the number of intercepted columns affects non-uniformity correction and provides guidance for selecting an optimal column count.

The experimental data consist of 40 frames of noise-free high-resolution infrared images with an original image size of 1024 × 55,000. Intercepted images of size 1024 × 8192 were used for evaluation. Streak noise was added, with the gain coefficient g(i) following a Gaussian distribution (mean = 1, variance = 0.02) and the bias coefficient b(i) following a Gaussian distribution (mean = 0, variance = 0.02). No additional Gaussian white noise was introduced.

The number of intercepted columns was incrementally increased from 400 to 3200, and correction parameters were calculated and applied to the full map. The correction effect was evaluated using the mean PSNR, as shown in Table 1.

As shown in Table 1, the PSNR value improves as the number of intercepted columns increases. Beyond 1600 columns, the PSNR stabilizes, indicating that the correction parameters effectively capture the noise characteristics of the entire map. Increasing the number of columns beyond 1600 yields less improvement (less than 0.1 in PSNR) while increasing computational overhead. Thus, selecting around 1600 columns for practical applications balances correction accuracy and computational efficiency.

### 3.3. Effect of Image Scene Complexity on Non-Uniformity Correction

**Figure 6:**
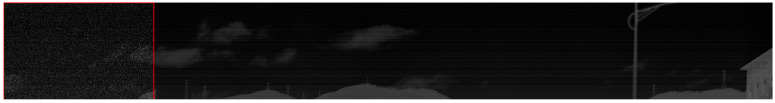
Long-wave infrared weekly scanning image with Gaussian white noise added to the left side, indicated by the red line.

To study the impact of scene complexity on calibration performance, this experiment simulates infrared line-scanning images by adding Gaussian white noise with varying variances. Simulated images of size 1024 × 8192 were used, with 1600 columns intercepted for correction parameter calculation. The gain g(i) and bias b(i) follow Gaussian distributions with means of 1 and 0 and variances of 0.02, respectively. The variance of the Gaussian white noise n(i,j) was gradually increased from 0.02 to 0.2 to simulate scenes of varying complexity. The experiment was conducted on 40 frames of noise-free, 14-bit infrared line-scanning images. An example of the noisy image after adding Gaussian noise is shown in Figure 6, and the PSNR values for corrected images under different noise variances are presented in Table 2.

From Table 2, the PSNR increases as the noise variance grows, reaching a peak at a variance of 0.1. This indicates that the algorithm effectively separates streak noise from the background while maintaining image structure under moderate noise levels. However, when the noise variance exceeds 0.1, the PSNR declines due to the increased noise intensity destroying local structural information, making it challenging for the algorithm to fully recover the original image details.

These results demonstrate that the algorithm is robust in varying scene complexities. It handles non-uniformity correction in complex scenarios, making it suitable for infrared line-scanning detectors under diverse conditions.

### 3.4. Quantitative Analysis of Algorithmic Non-Uniformity Correction

The algorithms in this paper are compared experimentally with state-of-the-art non-uniformity correction methods, including MSGF, 1DGF, GFLF, ENSI, and CNLM. To evaluate performance comprehensively, reference evaluation metrics such as PSNR, SSIM, and roughness are used in the simulated images. In contrast, non-reference metrics such as ICV and GC assess the correction effect on real images.

#### 3.4.1. Experimental Datasets

This study utilizes two types of experimental dataset: simulated and real.

Simulated datasets were created to compare streak noise removal models under controlled conditions. The FLIRADAS and MassMIND datasets serve as reference datasets. FLIRADAS is a thermal imaging dataset captured with a vehicle-mounted RGB thermal camera, containing 4224 infrared images at a resolution of 480 × 640. MassMIND is a long-wave infrared oceanographic dataset comprising 2916 images with a resolution of 512 × 640. From these datasets, 100 images were randomly selected, and five types of streak noise were added using Equation (21):

Case 1: g(i) and b(i) follow Gaussian distributions (mean = 1, variance = 0.02). No Gaussian white noise is added, simulating weak streak noise to test the algorithm’s correction ability under mild conditions.
Case 2: g(i) and b(i) follow Gaussian distributions (mean = 1, variance = 0.05). Moderate streak noise is simulated to evaluate correction under intermediate conditions.
Case 3: g(i) and b(i) follow Gaussian distributions (mean = 1, variance = 0.08). This case simulates strong streak noise, testing the algorithm’s performance under extreme conditions.
Case 4: gain noise varies linearly and periodically in the horizontal direction, simulating complex, non-uniform streak noise.
Case 5: g(i) and b(i) follow Gaussian distributions (mean = 1, variance = 0.02), with added Gaussian white noise n(i,j) (variance = 0.04) to simulate environmental interference and test robustness.

Two real datasets containing streak noise were also used for comparison: Tendero’s Dataset, which consists of 20 infrared images of varying resolutions, and the long wave infrared weekly scanning dataset, which contains 40 images with a resolution of 1024 × 55,000. For the experiments, 1024 × 8192 columns were intercepted from the long-wave dataset.

#### 3.4.2. Parameter Setting

All algorithms were run on the same set of infrared images to ensure a fair comparison under consistent pre-processing steps. Parameters were drawn from each method’s original references whenever possible, with minor modifications to accommodate this study’s resolutions and noise characteristics. Specifically, for our proposed method, we adopted a [15 × 1] window in the guided filtering stage to capture horizontal streak noise effectively, with a smoothing parameter r=0.16 balancing noise suppression and detail retention. The iterative compensation was capped at five cycles after pilot tests showed negligible PSNR/SSIM improvements beyond five iterations, and an initial compensation coefficient of α=0.05 was chosen to avoid over-smoothing. In contrast, MSGF [43] employed wavelet decomposition (sym8, three levels), followed by a guided filter of size [5 × 5] and a smoothing parameter ε=0.01. The 1DGF [23] used a one-dimensional guided filter with a [1 × 100] window and ε=0.01, reflecting its emphasis on row-wise filtering in low-texture infrared settings. For GFLF [22], we set an [8 × 1] horizontal filtering window and a [1 × 100] vertical filtering window, applying smoothing parameters of 0.04 horizontally and 0.16 vertically to address both row- and column-wise artifacts. ENSI [24] employed a Gaussian filter window of [1 × 5] after converting the image dimension from 2D to 1D. At the same time, CNLM [39] used a [7 × 7] search window for identifying similar patches and a similarity threshold of h^2^ = 0.1, thereby balancing fine detail preservation with effective noise removal. Notably, for the large-scale long-wave infrared weekly scanning dataset, our algorithm and GFLF extracted 1600 columns to capture the global noise distribution efficiently. All other parameters were consistent with their respective references, ensuring each algorithm operated near its recommended conditions for a fair and transparent performance evaluation.

#### 3.4.3. Parameter Sensitivity Analysis

To further address the choice of multiple parameters in the algorithm, we performed an ablation study on four key hyperparameters: (1) the guide filtering window size w, (2) the smoothing parameter r, (3) the maximum iteration count n, and (4) the initial compensation coefficient α. We selected 30 representative images from the FLIRADAS dataset (Case1 noise scenario) and systematically varied each parameter while keeping the others fixed at their default values.

**Figure 7:**
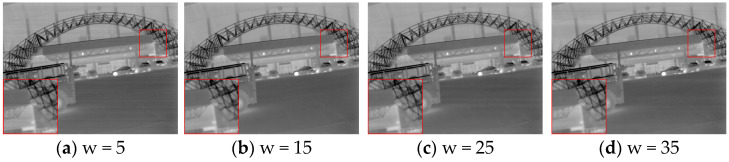
Comparison chart of correction for different guided filtering windows w.

The zoomed-in area in Figure 7 shows that a smaller window size [5 × 1] leaves noticeable streaks, while a larger window size [35 × 1] causes blurred edges. A medium-sized window size [15 × 1] can balance detail preservation and denoising.

**Figure 8:**
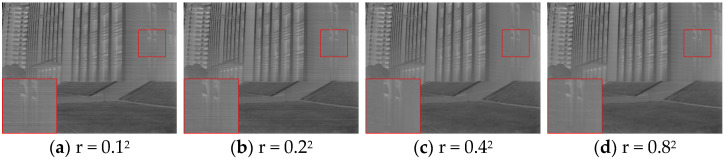
Comparison chart of correction for different smoothing parameters r.

From the enlarged area of Figure 8, we can see that when r = 0.1^2^ or r = 0.2^2^, the image is relatively smooth, and the details are relatively clear, but more noise is left. When r = 0.4^2^ or 0.8^2^, the noise removal effect is noticeable, but excessive smoothing leads to a loss of image details, especially when r = 0.8^2^, when the texture of the building becomes blurred.

**Figure 9:**
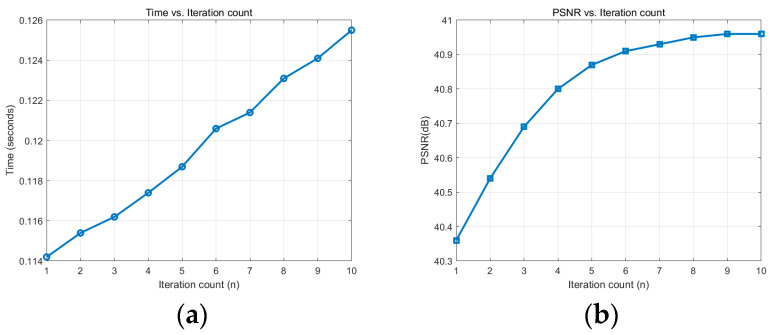
Time and PSNR under different iteration counts: (**a**) time vs. iteration count; (**b**) PSNR vs. iteration count.

According to Figure 9, as the number of iterations n increases, the PSNR gradually increases, but the increase decreases while the time increases linearly. Considering the performance and computing time, it is ideal to choose n = 5 to n = 7 because, in this range, the improvement of PSNR has stabilized, and the running time is relatively reasonable.

**Figure 10:**
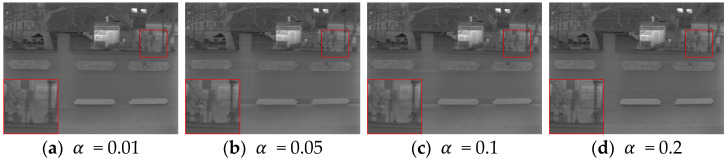
Comparison chart of correction for different initial compensation coefficient α.

As can be seen from Figure 10, when α = 0.2, the correction will overshoot, and when α is small, more iterations are required to achieve the same effect. Between 0.05 and 0.1, the convergence speed and correction quality are better balanced.

Based on these observations, we suggest using [15 × 1] for the guide window, r = 0.16, up to five iterations, and α = 0.05 as default. Nevertheless, users can fine-tune these parameters if confronted with significantly different imaging conditions or noise intensities.

#### 3.4.4. Evaluation Indicators

Several evaluation metrics are employed to assess the performance of the algorithms:

PSNR evaluates the similarity between the corrected image and the original image. A higher PSNR indicates better correction and detail retention. The formula is as follows:

(22) $$PSNR=10\log_{10}\left(\frac{MAX^{2}}{MSE}\right)$$

where MAX is the maximum possible pixel value of the image and MSE represents the mean squared error between the corrected and reference images.

SSIM measures the brightness, contrast, and structure similarity between the corrected and original images. The closer the SSIM value is to 1, the higher the subjective quality. The formula is as follows:

(23) $$SSIM=\frac{\left(2\mu_{\hat{I}}\mu_{I}+C_{1}\right)\left(2\sigma_{\hat{I}I}+C_{2}\right)}{\left(\mu_{\hat{I}}^{2}+\mu_{I}^{2}+C_{1}\right)\left(\sigma_{\hat{I}}^{2}+\sigma_{I}^{2}+C_{2}\right)}$$

where $\mu_{I}$, $\mu_{\hat{I}}$, $\sigma_{I}^{2}$, and $\sigma_{I}^{2}$ are the mean and variance of I and $\hat{I}$, respectively; $\sigma_{\hat{I}I}$ is the covariance between I and $\hat{I}$; and $C_{1}$ and $C_{2}$ are constants to avoid division by zero.

Roughness is used to measure the overall frequency characteristics of an image. The closer the roughness value of the corrected image is to that of the original image, the better the correction effect. The calculation formula is as follows:

(24) $$p=\frac{\left\|h*\hat{I}\right\|+\left\|h^{T}*I\right\|}{\left\|I\right\|}$$

where ||·|| denotes the L-1 norm, ∗ denotes the convolution operation, h is the horizontal gradient operator, $\hat{I}$ is the corrected image, $h^{T}$ is the vertical gradient operator, and I is the input image.

ICV assesses uniformity and contrast enhancement by measuring the ratio of the mean to the standard deviation of the pixel values in selected regions. A higher ICV indicates better uniformity. The formula is as follows:

(25) $$ICV=\frac{{\hat{I}}_{m}}{{\hat{I}}_{S}}$$

where ${\hat{I}}_{m}$ and ${\hat{I}}_{S}$ are the mean and standard deviation of the selected region.

GC reflects the retention of image details by comparing the gradients of the corrected and original images. A smaller GC value indicates higher detail preservation. The formula is as follows:

(26) $$GC=\frac{\Sigma \left|Grad\left(I\right)-Grad\left(\hat{I}\right)\right|}{\Sigma Grad\left(I\right)}$$

where Grad(·) denotes the gradient calculation of the image, I is the input image, and $\hat{I}$ is the output image.

#### 3.4.5. Quantitative Testing of Simulated Datasets

The proposed algorithm’s performance was evaluated using simulated datasets derived from FLIRADAS and MassMIND, incorporating five types of streak noise. The proposed method was compared against the MSGF, 1DGF, GFLF, ENSI, and CNLM algorithms regarding PSNR, SSIM, and roughness. The results are summarized in Table 3. To provide a clearer and more focused comparison, the row-mean images from these algorithms are shown in Figure 7 and Figure 8, with each case displayed separately for better clarity.

**Figure 11:**
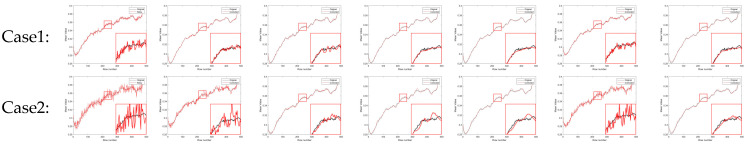

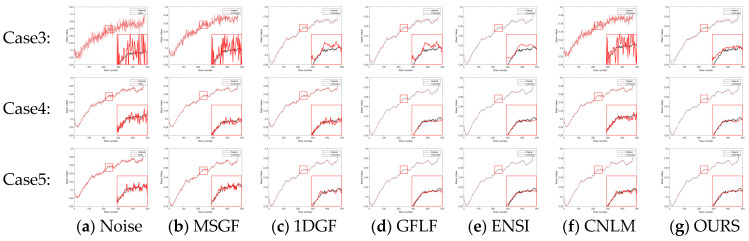
Row-mean images of different algorithms on the FLIRADAS dataset.

**Figure 12:**
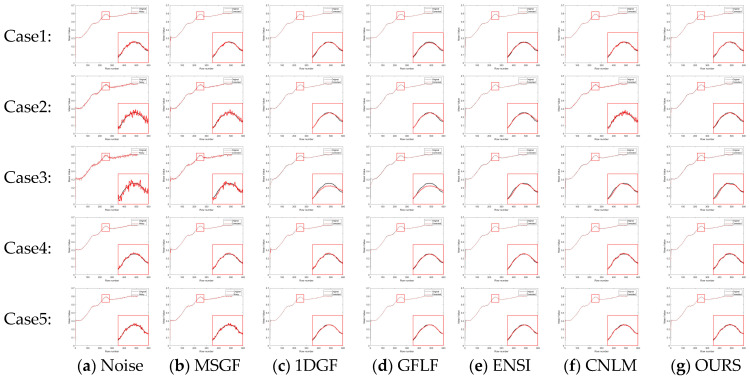
Row means of different algorithms on the MassMIND dataset.

In Figure 11a–g and Figure 12a–g, each row represents a specific noise level, and each column corresponds to a different algorithm. We show the row-mean images of different algorithms under various noise conditions. These figures clearly show that the proposed method outperforms other methods, especially in suppressing noise without losing key image details, where our proposed algorithm consistently performs the best under all noise levels.

In contrast, existing methods exhibited clear limitations. MSGF and 1DGF could not handle complex noise patterns effectively, often losing high-frequency details or introducing ripple artifacts. GFLF balanced denoising and detail retention but encountered artifacts due to its region division strategy. ENSI performed adequately for simple noise but failed in scenarios with higher noise intensity, while CNLM managed small-scale noise but struggled to recover details in larger-scale noise regions. The proposed method demonstrated superior adaptability and robustness in scenarios with significant challenges, such as Case4 and Case5, making it a reliable choice for complex noise conditions.

For further illustration, we zoom in on specific regions of the row-mean images obtained by different algorithms. In addition, the overall comparison in Table 3 provides a quantitative analysis of PSNR, SSIM, and roughness metrics, highlighting the superior performance of our method.

#### 3.4.6. Quantitative Testing of Real Datasets

To further evaluate the effectiveness of the proposed algorithm, experiments were conducted on real datasets, including Tendero’s dataset and the long-wave infrared weekly scanning dataset. These datasets contain more complex noise characteristics and inhomogeneities than simulated datasets, providing a more realistic assessment of the algorithm’s performance in practical applications. Table 4 summarizes the results for Tendero’s dataset, while Table 5 presents the long-wave infrared weekly scanning dataset results.

For Tendero’s dataset, the proposed algorithm achieves an ICV value of 2.3522, which is significantly higher than other algorithms, indicating superior contrast uniformity and effective suppression of streak noise. Additionally, the GC value of 0.0015, the lowest among all of the algorithms, reflects better retention of gradient details and avoidance of over-smoothing. By comparison, the MSGF and GFLF algorithms perform poorly in complex noise regions, losing details and resulting in lower ICV and higher GC values.

The proposed algorithm consistently outperforms alternatives in the long-wave infrared weekly scanning dataset. It achieves a PSNR of 43.21 dB and an SSIM of 0.9439, indicating enhanced denoising and structure preservation. While GFLF and 1DGF demonstrate reasonable performance, they suffer from detail loss and insufficient correction in high-noise regions, resulting in slightly lower PSNR and SSIM values. The algorithm’s ability to achieve an ICV value of 2.8053, the highest among all of the methods, further underscores its capability to provide uniform and high-quality correction for complex real-world infrared images.

#### 3.4.7. Analysis of the Quantitative Results of the Simulated Dataset

Four simulated datasets evaluated the proposed algorithm’s non-uniform correction capability. The KAIST dataset has 8995 images with a resolution of 512 × 640, which contain rich urban dynamic environments and day and night scenes and can complement the FLIRADAS dataset. The LLVIP dataset provides many low-light night scenes, consisting of 3463 images with a resolution of 1024 × 1280. One hundred images were randomly selected from each of the four simulated datasets. Noise was added to these images using Formula (21) for further testing.

**Figure 13:**
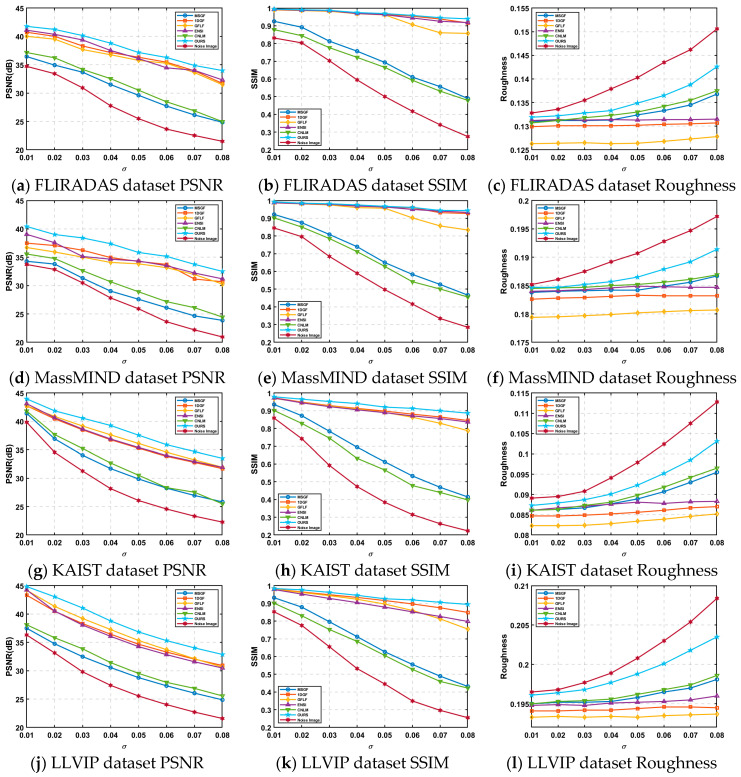
Test results for four datasets.

As evident from Figure 13, the proposed algorithm consistently achieves higher PSNR and SSIM values across all noise scenarios, particularly excelling in high-noise conditions. This indicates its robust ability to suppress noise while preserving structural details and overall image quality. By leveraging residual-based compensation and weighted linear regression, the algorithm addresses the challenges of detail loss and uneven correction faced by traditional methods.

Overall, the algorithm demonstrates superior adaptability and effectiveness across both simulated and real datasets, establishing itself as a reliable solution for denoising high-resolution infrared images under complex noise conditions.

### 3.5. Qualitative Analysis of the Effect of Algorithmic Non-Uniformity Correction

#### 3.5.1. Qualitative Analysis of Simulated Data

**Figure 14:**
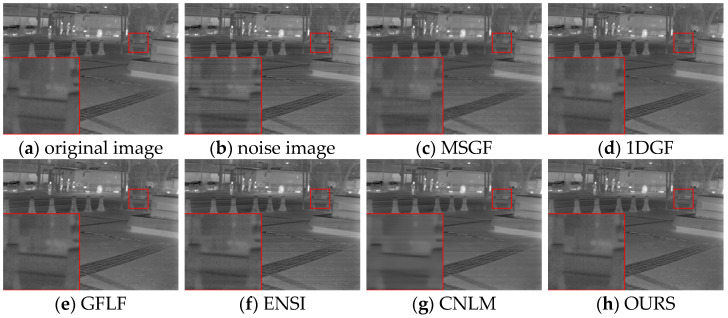
Correction plots of different algorithms for FLIRADAS dataset under Case1 noise.

Under varying noise conditions, different algorithms’ denoising performance and detail preservation were evaluated qualitatively. As shown in Figure 14, under weaker noise conditions, the MSGF algorithm leaves behind noticeable streak noise in certain regions, failing to preserve finer image details. The 1DGF algorithm effectively suppresses noise but introduces a degree of over-smoothing, particularly evident in the blurring of image edges and structural information. Conversely, the GFLF algorithm provides better noise suppression but sacrifices some texture fidelity due to excessive smoothing in complex structural areas. The ENSI algorithm exhibits high background smoothness but struggles with local uniformity and fails to address noise consistently. The CNLM method retains more texture details but leaves residual streak noise, reflecting an imbalance between noise suppression and detail retention. In comparison, the proposed algorithm achieves a superior balance, effectively removing streak noise while maintaining texture integrity, particularly in regions of structural complexity.

**Figure 15:**
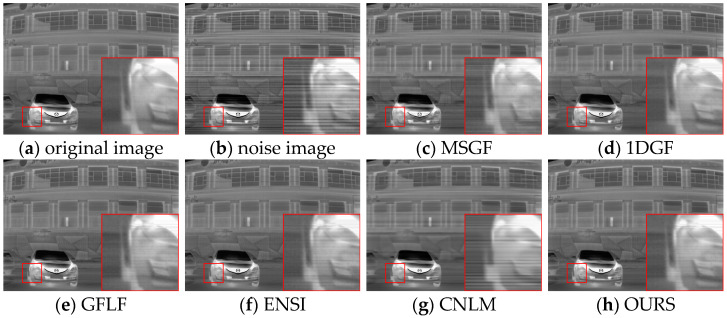
Correction plots of different algorithms for FLIRADAS dataset under Case2 noise.

For simulated datasets, including the FLIRADAS and MassMIND datasets, the proposed algorithm demonstrated robustness in addressing non-uniformity and preserving essential image features under various noise intensities. As depicted in Figure 15, our algorithm effectively removes horizontal stripe noise under moderate noise conditions while maintaining detailed texture information, such as structural contours and edge clarity. In contrast, the MSGF and CNLM algorithms leave significant noise artefacts, particularly in low-contrast areas, while the 1DGF algorithm struggles with over-smoothing. The red-boxed areas show that the GFLF and ENSI methods provide acceptable denoising performance but lose details in complex image regions.

**Figure 16:**
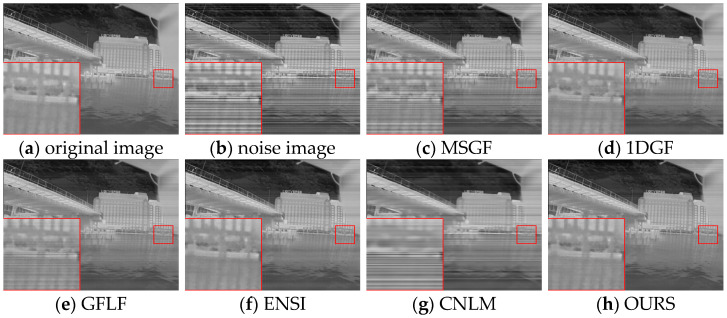
Correction plots of different algorithms for MassMIND dataset under Case3 noise.

Figure 16 illustrates the calibration results for Case3 noise intensity using the MassMIND dataset. Here, the GFLF algorithm exhibits a more apparent trade-off between stripe noise removal and texture preservation, with noticeable blurring in high-noise regions. The CNLM algorithm fails to adapt to the complex noise distribution fully, leaving residual noise streaks. However, the proposed algorithm achieves a robust balance by leveraging local weight fusion and adaptive smoothing mechanisms, delivering visually consistent results with improved detail retention.

#### 3.5.2. Qualitative Analysis of Real Data

**Figure 17:**
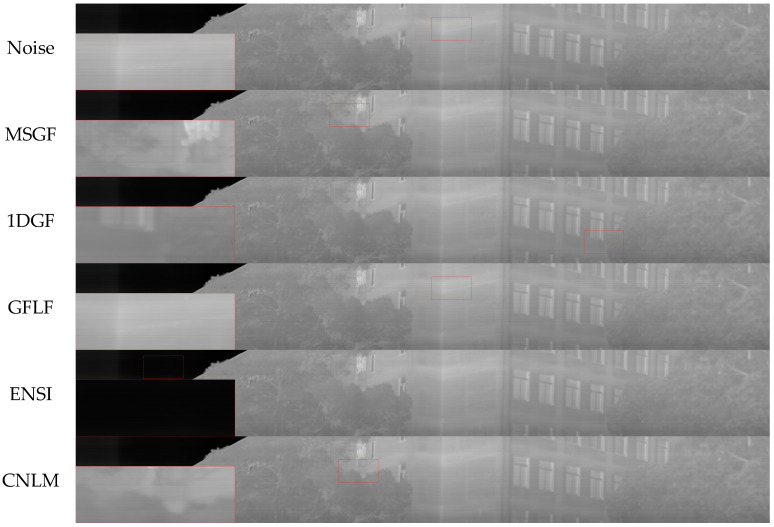

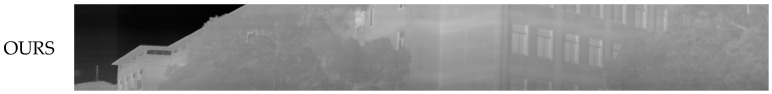
Corrected plots of long-wave infrared weekly scanning images in different algorithms.

As observed in Figure 17, the MSGF method shows poor denoising performance, with prominent horizontal stripe noise remaining due to the loss of structural information caused by the wavelet transform. The 1DGF algorithm exhibits blurred details in the tree region on the right side and slight brightness unevenness in the transition between the left and right areas. The GFLF method leaves horizontal texture residues on the building wall, where noise blends with structural details. ENSI displays noticeable horizontal noise in the sky region on the left, attributed to inadequate handling of low-frequency backgrounds during smoothing, resulting in residual noise and a blurred tree texture. Similarly, the CNLM method leaves residual noise in the sky and flat building areas, with uneven brightness at the boundary between the sky and trees. In contrast, the OURS algorithm effectively suppresses horizontal stripe noise while preserving image details, particularly along building edges and tree contours. The noise is smoothed while retaining clear structural information without introducing significant blurring.

### 3.6. Algorithm Time Complexity Analysis

To analyze the time efficiency of our proposed algorithm, experiments were conducted on real high-resolution infrared line-scanning images with dimensions of 1024 × 55,000 pixels and 14-bit acquisition accuracy. Table 6 compares the average runtime of 10 executions for our algorithm against WAGE [21], 1DGF [22], GFLF [23], ADOM [28], TV-STV [29], CNLM [39], and MSGF [43].

As shown in Table 6, our algorithm achieves a runtime of 1.6821 s, which is competitive with GFLF (1.5114 s) and significantly faster than other methods like ADOM and FTV-STV, which require over 180 and 887 s, respectively. The lightweight iterative compensation and efficient guide filtering contribute to this advantage. In comparison, the IDGF method also demonstrates reasonable performance with a runtime of 5.2821 s, but its processing involves computational overhead for row and column guide filtering. Other methods, such as MSGF and WAGE, rely on multiscale or high-dimensional processing, resulting in significantly longer runtimes.

To further evaluate the efficiency of our algorithm, Table 7 and Table 8 present detailed performance metrics for varying numbers of intercepted columns during the correction process. Our algorithm demonstrates a steady increase in runtime yet maintains high efficiency with less than 2 s required for up to 6000 columns. Similarly, the PSNR and SSIM metrics remain consistent and robust across different column intercepts, achieving average values of 36.97 dB and 0.8685 at the final iteration.

Table 9 and Table 10 detail the performance for selected scenarios with 5000 and 6000 columns, where N represents the number of iterations. Our algorithm consistently achieves superior PSNR and SSIM metrics compared to the GFLF algorithm while maintaining minimal computational overhead. For instance, our algorithm reports a PSNR of 36.84 dB and an SSIM of 0.8642 for 6000 columns, clearly outperforming GFLF’s performance under the same conditions.

The proposed algorithm effectively balances computational efficiency and correction quality, demonstrating significant advantages in real-time processing scenarios, particularly for high-resolution infrared line-scanning images.

## 4. Discussion

This study introduces a non-uniformity correction method tailored for high-resolution infrared line-scanning images, leveraging residual guidance and adaptive weighting. The proposed framework addresses challenges such as directional stripe noise, loss of detail, and stringent real-time processing requirements. Our method demonstrates significant improvements in denoising performance while maintaining high levels of detail preservation, which is crucial for infrared imaging applications, especially those involving complex, non-uniform noise patterns.

The proposed algorithm effectively combines residual guided filtering and adaptive weighting techniques. Utilizing both residual and original images optimizes preserving fine details while smoothing the noise in a controlled manner. This dual-guidance mechanism helps overcome the edge-blurring issues typically associated with traditional guided filtering methods. The adaptive compensation mechanism further enhances performance by dynamically adjusting the compensation strength, thus preventing over-correction and ensuring that critical image details are retained during noise suppression.

Moreover, using weighted linear regression based on local variance allows the algorithm to adapt to varying noise intensities across different image regions. This localized correction ensures a more accurate global correction, especially in complex and noisy regions. In contrast, conventional methods that rely on uniform correction parameters often struggle with maintaining detail in areas with varying noise levels.

Extensive experimental results, both quantitative and qualitative, confirm that our algorithm outperforms existing methods in terms of key performance metrics, such as PSNR, SSIM, and roughness. Our method achieves superior noise suppression and preserves the image’s structural integrity across multiple datasets, including simulated and real infrared images. In particular, the algorithm excels in scenarios with higher noise intensities and more complex noise patterns, demonstrating its robustness and adaptability to challenging conditions.

One key advantage of our approach is its ability to meet real-time processing requirements without compromising the quality of the correction. The proposed algorithm achieves efficient correction with minimal computational overhead, making it suitable for real-time infrared imaging systems applications. This efficiency, combined with the high-quality denoising performance, ensures that the algorithm can be effectively used in dynamic environments where high resolutions and fast processing are essential.

Despite the strengths of our method, some areas could benefit from further improvement. The current method assumes a Gaussian noise model, which may not fully capture the complexities of real-world noise, particularly in more dynamic environments. Future work could incorporate more advanced noise models to handle a broader range of noise types. Additionally, using parallel computing techniques or hardware accelerators, such as GPUs or FPGAs, could further enhance the real-time processing capabilities of the algorithm, allowing it to handle even larger datasets more efficiently.

In conclusion, the proposed algorithm represents a robust and efficient solution for non-uniformity correction in high-resolution infrared line-scanning images. It significantly improves existing methods by addressing the challenges of directional stripe noise and maintaining fine detail. Its adaptability to complex noise conditions and real-time performance make it a promising tool for various infrared imaging applications, including environmental monitoring, military surveillance, and thermal imaging.

## Figures and Tables

**Table 1:** Mean PSNR values of corrected images with different numbers of intercepted columns.

**Columns**	400	800	1200	1600	2000	2400	2800	3200
**PSNR**	42.02	42.13	42.28	42.46	42.47	42.47	42.49	42.5

**Table 2:** Effect of different Gaussian white noises on calibration effect.

**variance**	0.02	0.04	0.06	0.08	0.1
**PSNR**	42.86	43.28	44.06	44.04	44.18
**variance**	0.12	0.14	0.16	0.18	0.2
**PSNR**	43.95	43.59	43.27	43.09	42.62

**Table 3:** Quantitative test results for the simulated dataset.

Simulated Image Data		Metric	Noise	MSGF	1DGF	GFLF	ENSI	CNLM	OURS
FLIRADAS Data		PSNR	33.43	34.94	40.02	39.52	40.37	36.23	41.84
	Case1	SSIM	0.8035	0.8918	0.9871	0.9859	0.9876	0.8442	0.9905
		Roughness	0.1336	0.1312	0.1301	0.1264	0.1314	0.1312	0.1322
		PSNR	25.5	29.62	36.33	35.78	36.1	30.52	37.87
	Case2	SSIM	0.5005	0.6933	0.9655	0.9616	0.9629	0.6652	0.9691
		Roughness	0.1403	0.1324	0.1302	0.1264	0.1313	0.1338	0.1349
		PSNR	21.5	24.85	31.71	31.48	32.33	24.97	34.58
	Case3	SSIM	0.2747	0.4896	0.9164	0.8572	0.9182	0.4791	0.9396
		Roughness	0.1506	0.1368	0.1307	0.1278	0.1315	0.1395	0.1426
		PSNR	34.01	35.21	40.36	39.72	41.05	35.52	41.98
	Case4	SSIM	0.8206	0.8992	0.9881	0.9867	0.9903	0.8523	0.9948
		Roughness	0.1336	0.1313	0.1303	0.1265	0.1316	0.1313	0.1323
		PSNR	26.94	28.13	27.84	28.16	27.88	27.94	28.72
	Case5	SSIM	0.457	0.5186	0.5006	0.5208	0.5018	0.5035	0.5318
		Roughness	0.1394	0.1356	0.1360	0.1322	0.1372	0.1293	0.1382
MassMIND Data	Case1	PSNR	32.85	33.8	37.07	35.93	37.57	34.78	39.01
		SSIM	0.7961	0.8749	0.9827	0.9811	0.9826	0.8505	0.9854
		Roughness	0.1861	0.184	0.1828	0.1795	0.1841	0.1846	0.1847
	Case2	PSNR	25.91	27.59	34.26	33.84	34.34	28.91	35.85
		SSIM	0.4974	0.6501	0.9626	0.9567	0.9637	0.6282	0.9676
		Roughness	0.1907	0.1842	0.1833	0.1802	0.1849	0.1852	0.1865
	Case3	PSNR	20.94	23.87	30.68	30.27	31.19	24.45	32.51
		SSIM	0.2848	0.4661	0.9270	0.8339	0.9332	0.4556	0.9488
		Roughness	0.1972	0.1867	0.1832	0.1807	0.1847	0.1869	0.1914
	Case4	PSNR	34	34.52	37.38	36.03	38.01	35.66	39.18
		SSIM	0.8222	0.9001	0.9839	0.9818	0.9852	0.8696	0.9885
		Roughness	0.1859	0.1840	0.1829	0.1797	0.1846	0.1846	0.1848
	Case5	PSNR	26.75	27.83	27.48	27.96	27.5	27.72	28.51
		SSIM	0.4483	0.5046	0.4918	0.5198	0.4931	0.4948	0.5462
		Roughness	0.1903	0.1872	0.1871	0.1839	0.1889	0.1832	0.1891

**Table 4:** Test results for Tendero’s dataset.

Real Image Data	Metric	Noise	MSGF	1DGF	GFLF	ENSI	CNLM	OURS
Tendero’s data	ICV	1.9189	1.9447	2.2653	2.1321	2.3026	2.2831	2.3522
	GC	---	0.2383	0.0156	0.0434	0.0054	0.6664	0.0015

**Table 5:** Test results for the long-wave infrared weekly scanning dataset.

Real Image Data	Metric	Noise	MSGF	1DGF	GFLF	ENSI	CNLM	OURS
Long-wave infrared weekly scanning dataset	PSNR	----	39.75	40.19	39.81	40.1	39.46	43.21
	SSIM	---	0.9053	0.9176	0.9149	0.9161	0.9122	0.9439
	Roughness	0.0932	0.0924	0.0925	0.0923	0.0925	0.0923	0.0929
	ICV	2.6684	2.6954	2.7078	2.7211	2.7072	2.7032	2.8053
	GC	---	0.2641	0.0006	0.0163	0.0005	0.0184	0.0002

**Table 6:** Comparison of time complexity of different algorithms.

**Algorithms**	MSGF	1DGF	GFLF	ADOM	CNLM	WAGE	FTV-STV	OURS
**Time/s**	6.8603	5.2821	1.5114	185.0071	415.1505	313.1802	887.5803	1.6821

**Table 7:** GFLF intercepts different columns of statistical results.

**Columns**	5000	6000	7000	8000	9000	10,000
**Time/s**	1.5114	1.6827	1.8745	2.0271	2.2164	2.3832
**PSNR**	34.17	34.18	34.26	34.26	34.25	34.25
**SSIM**	0.7652	0.7658	0.767	0.767	0.767	0.7672

**Table 8:** Our algorithm intercepts different columns of statistical results.

**Columns**	5000	6000	7000	8000	9000	10,000
**Time/s**	1.6821	1.8783	2.0713	2.2639	2.4212	2.6001

**Table 9:** Our algorithm intercepts 5000 columns of statistical results.

**N**	1	2	3	4	5
**Time/s**	1.6209	1.6334	1.6447	1.6647	1.6821
**PSNR**	36.32	36.45	36.57	36.68	36.77
**SSIM**	0.8462	0.851	0.8551	0.8586	0.8617

**Table 10:** Our algorithm intercepts 6000 columns of statistical results.

**N**	1	2	3	4	5
**Time/s**	1.7661	1.8056	1.8201	1.8458	1.8783
**PSNR**	36.31	36.47	36.61	36.73	36.84
**SSIM**	0.8462	0.8518	0.8566	0.8607	0.8642

## Data Availability

An infrared long-wave cooled linear scan detector generated the real infrared image dataset. It is not a public dataset. The publicly available dataset FLIRADAS was analyzed in this study and can be found here: https://camel.ece.gatech.edu/, accessed on 12 December 2024. The publicly available dataset MassMIND was analyzed in this study and can be found here: https://github.com/uml-marine-robotics/MassMIND, accessed on 12 December 2024. The publicly available Tendero dataset was analyzed in this study and can be found here: https://ipolcore.ipol.im/demo/clientApp/demo.html?id=129, accessed on 12 December 2024. The publicly available dataset LLVIP was analyzed in this study and can be found here: https://github.com/bupt-ai-cz/LLVIP, accessed on 18 February 2025. The publicly available dataset KSIST was analyzed in this study and can be found here: https://soonminhwang.github.io/rgbt-ped-detection/, accessed on 18 February 2025.
